# High Avidity Antibodies to Full-Length VAR2CSA Correlate with Absence of Placental Malaria

**DOI:** 10.1371/journal.pone.0040049

**Published:** 2012-06-26

**Authors:** Yeung Lo Tutterrow, Ali Salanti, Marion Avril, Joseph D. Smith, Ian S. Pagano, Simon Ako, Josephine Fogako, Rose G. F. Leke, Diane Wallace Taylor

**Affiliations:** 1 Department of Tropical Medicine, Medical Microbiology and Pharmacology, John A. Burns School of Medicine, University of Hawai'i-Mānoa, Honolulu, Hawaii, United States of America; 2 Department of International Health, Immunology and Microbiology, Centre for Medical Parasitology, University of Copenhagen and Department of Infectious Diseases, Copenhagen University Hospital (Rigshospitalet), Copenhagen, Denmark; 3 Seattle Biomedical Research Institute, Seattle, Washington, United States of America; 4 Cancer Research Center, University of Hawaii-Mānoa, Honolulu, Hawaii, United States of America; 5 The Biotechnology Center, University of Yaoundé I, Yaoundé, Cameroon; Royal Tropical Institute, The Netherlands

## Abstract

VAR2CSA mediates sequestration of *Plasmodium falciparum*-infected erythrocytes in the placenta, increasing the risk of poor pregnancy outcomes. Naturally acquired antibodies (Ab) to placental parasites at delivery have been associated with improved pregnancy outcomes, but Ab levels and how early in pregnancy Ab must be present in order to eliminate placental parasites before delivery remains unknown. Antibodies to individual Duffy-binding like domains of VAR2CSA have been studied, but the domains lack many of the conformational epitopes present in full-length VAR2CSA (FV2). Thus, the purpose of this study was to describe the acquisition of Ab to FV2 in women residing in high and low transmission areas and determine how Ab levels during pregnancy correlate with clearance of placental parasites. Plasma samples collected monthly throughout pregnancy from pregnant women living in high and low transmission areas in Cameroon were evaluated for Ab to FV2 and the proportion of high avidity Ab (i.e., Ab that remain bound in the presence of 3M NH_4_SCN) was assessed. Ab levels and proportion of high avidity Ab were compared between women with placental malaria (PM^+^) and those without (PM^−^) at delivery. Results showed that PM^−^ women had significantly higher Ab levels (p = 0.0047) and proportion of high avidity Ab (p = 0.0009) than PM^+^ women throughout pregnancy. Specifically, women with moderate to high Ab levels (>5,000 MFI) and those with ≥35% high avidity Ab at 5–6 months were found to have 2.3 (95% CI, 1.0–4.9) and 7.6-fold (p = 0.0013, 95% CI: 1.2–50.0) reduced risk of placental malaria, respectively. These data show that high levels of Ab to FV2, particularly those with high avidity for FV2, produced by mid-pregnancy are important in clearing parasites from the placenta. Both high Ab levels and proportion of high avidity Ab to FV2 may serve as correlates of protection for assessing immunity against placental malaria.

## Introduction

*Plasmodium falciparum* infections increase the risk of maternal anemia, delivery-related complications and poor pregnancy outcomes [1]–[5]. A major reason for the severity of malaria during pregnancy is the binding of infected erythrocytes (IE) via VAR2CSA to chondroitin sulfate A (CSA) on syncytiotrophoblasts and extracellular matrix in the intervillous space of the placenta [6]–[9]. As a result, IE sequester in the placenta causing placental malaria (PM). After one or more pregnancies, women develop antibodies (Ab) to VAR2CSA that improve pregnancy outcomes, including reduced risk of maternal anemia [10], [11], low birth weight babies [8], [12], and placental parasitemia [13]. *In vitro* and *ex vivo* studies show that naturally acquired Ab block IE from binding to placenta tissue and CSA [14]–[16] and promote phagocytosis by macrophages [17]–[19]; however, characteristics of the Ab response required for women to clear placental parasites remains unclear. It is also unknown how development of Ab to VAR2CSA differs between women living in high versus low transmission areas. Answers to these questions will provide crucial information for the forthcoming VAR2CSA clinical trials and for assays to evaluate protection from placental malaria.

In this study, the acquisition of Ab specific for full-length VAR2CSA (FV2) was determined using plasma collected longitudinally from women living in areas of high and low perennial malaria transmission, prior to implementation of intermittent presumptive treatment and insecticide-treated bednets. This is the first study to report the natural acquisition of Ab to all Duffy-binding like (DBL) domains, as previous studies have measured either the binding of Ab to the surface of IE and/or to recombinant DBL domains of VAR2CSA [20]–[24]. It is important to examine naturally acquired Ab to FV2, since individual DBL domains exhibit lower specificity and binding affinity for CSA compared to full-length VAR2CSA [25], [26]; while, measuring Ab to the surface of IE is less specific compared to Ab responses to recombinant FV2.

In previous studies, an association between inhibition of binding and clearance of parasites from the intervillous space of the placenta was not found using serum samples collected at delivery [27], [28]. Here we sought to determine if Ab levels at the end of the first, during the second, and/or third trimesters correlated with clearance of placental infections by comparing the responses of women who had placental malaria (PM^+^) and those without (PM^−^) at delivery. Furthermore, since immune exposure to VAR2CSA is primarily pregnancy-associated, the restricted exposure raises the questions of whether and how soon women produce high avidity Ab to VAR2CSA (i.e., Ab with strong-binding to FV2). High avidity Ab resulting from affinity maturation are often correlated with strong activities against viruses [29], [30] and bacteria [31], as well as protection from diseases [30]. It was recently reported that individuals residing in malaria endemic areas with high affinity Ab to *P. falciparum* merozoite surface protein-2 (MSP-2) had prolonged periods without clinical malaria [32]. Accordingly, we also investigated the importance of high avidity anti-VAR2CSA Ab in clearing parasites of the placental phenotype.

## Results

### Description of Women

The composition of women in Ngali II (n = 39) and Yaoundé (n = 50) included in this study were similar with respect to age, length of pregnancy, and proportion of primigravidae (Table 1). Significantly more women in Ngali II became slide-positive during pregnancy (p = 0.01), but the prevalence of slide-positivity did not differ significantly between the two sites when analyzed by trimesters.

**Table 1:** Characteristics of women followed longitudinally

	Ngali II	Yaoundé	p values
Number of women	39	50	
Age (years)	23.2±5.4^a^	25.0±5.1	0.117
Length of pregnancy (weeks)	39.0±2.5^a^	39.5±1.7	0.312
Primigravidae (%)	33.3%	34.0%	1.000
Proportion of women who were			
Slide-positive 1 or more times^b^	79.5%	54.0%	0.01
PCR-positive one or more times^b^	100%	100%	
Percent (%) Slide positivity at			
3 Months of Pregnancy	36.8%	14.0%	0.087
4–6 Months of Pregnancy	36.3%	23.4%	0.299
7–9 Months of Pregnancy	34.6%	21.6%	0.197
Placental malaria-positive	51.9%	31.3%	0.090

a mean ± SD.

b samples were selected from women who were slide and/or PCR-positive at least once during pregnancy confirming that women had become infected.

### Acquisition of IgG to Full-length VAR2CSA

**Figure 1:**
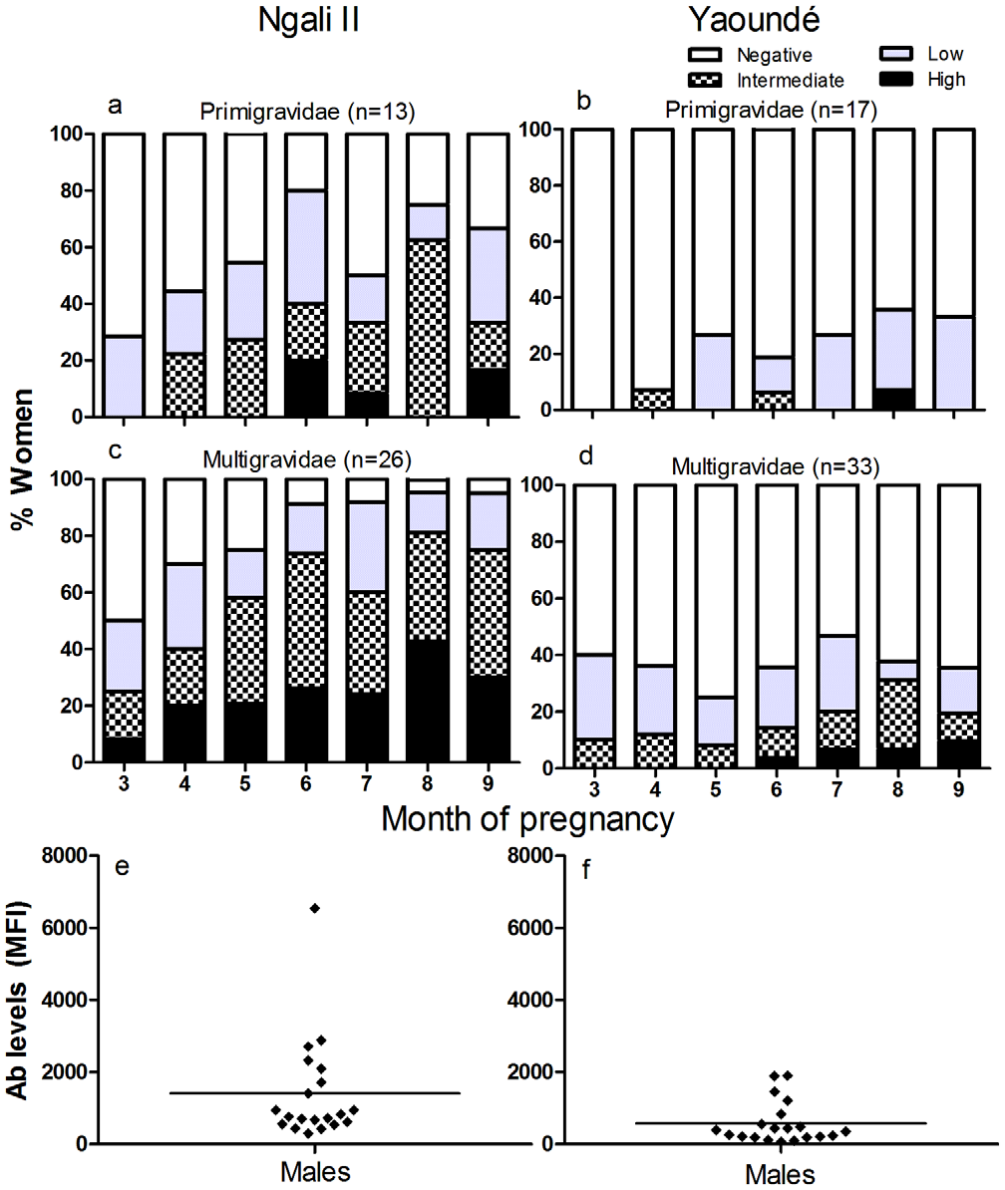
Natural Acquisition of IgG Antibodies to VAR2CSA. (**a–d**) Ab levels to FV2 were determined using plasma from (**a, b**) primigravidae and (**c, d**) multigravidae in Ngali II and Yaoundé. The total number of samples evaluated per month ranged from 19–37 for Ngali II and 39–46 for Yaoundé. Ab levels were divided into 4 categories: negative (white; below cutoff: the mean +2 SD of adult males in the same site), low (grey; above cutoff, but <5000 MFI), intermediate (checkered; 5,000–10,000 MFI) and high (black; >10,000 MFI). The percentage of women within each Ab group is shown. Women with Ab levels ≥5,000 MFI were considered to have strong Ab responses. (**e–f**) In comparison to pregnant women, only 1/40 male who lived in the two study sites had intermediate and 6/40 had low Ab levels to FV2. Antibody cutoff values for Ngali II and Yaoundé women were 2756 and 1192 MFI, respectively.

For ease, Ab responses were divided into four categories: negative (below cut-off), low (above cut-off, but <5,000 MFI), intermediate (5,000–10,000 MFI) and high (>10,000 MFI). Women with intermediate to high Ab levels were considered to have strong Ab responses. Cut-off values were defined by the mean+2 SD from corresponding endemic male controls (Fig. 1e–f). Acquisition of Ab to FV2 differed in women living in Ngali II and Yaoundé (Fig. 1). At 3 months of pregnancy, 28% of primigravidae in Ngali II had Ab (Fig. 1a); whereas, none of the primigravidae in Yaoundé were Ab-positive (Fig. 1b). By 4 months, 20% of primigravidae in Ngali II had developed strong responses (i.e., MFI >5,000); whereas, almost none of the primigravidae in Yaoundé developed strong responses prior to delivery. Likewise, multigravidae in Ngali II had more rapid responses than multigravidae in Yaoundé (Fig. 1c – d) and a higher proportion of multigravidae in Ngali II produced strong Ab responses at 6 (p<0.0001) and 8 months (p = 0.0006) than multigravidae in Yaoundé. Clearly, malaria transmission strongly influenced the acquisition of Ab to FV2.

The response to FV2 was predominantly pregnancy-associated as adult males living in two sites had very low reactivity with FV2 (Fig. 1e–f). Only 1 of 40 (2.5%) males had intermediate Ab levels and 6/40 (15%) had low levels at both sites. Thus, it is likely that most pregnant women with low Ab to FV2 seroconverted during pregnancy.

### Correlation between Levels of Antibodies to FV2 and Absence of Placental Malaria

**Figure 2:**
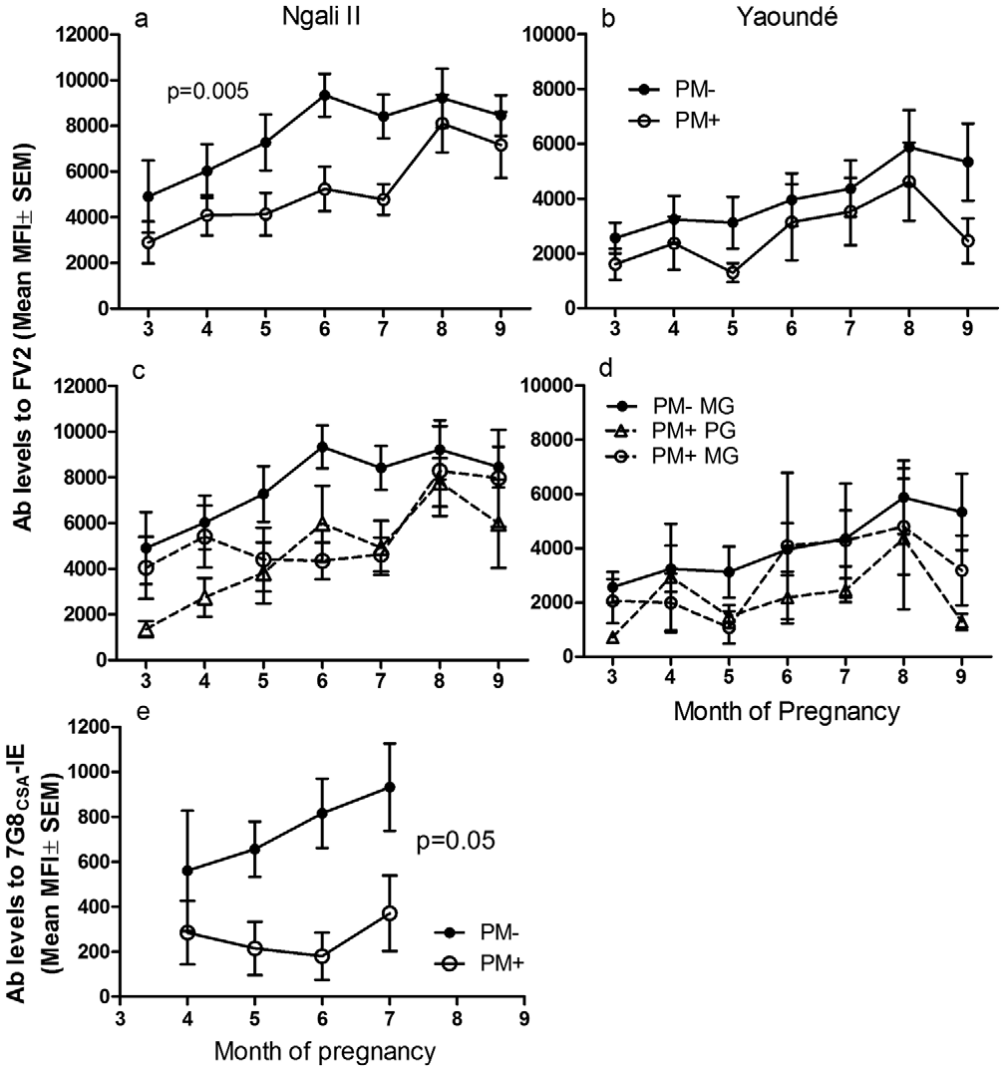
Correlation between Antibody Levels to VAR2CSA and Placental Malaria at Delivery. Anti-FV2 Ab levels (MFI ± SEM) were determined between 3 to 9 months of pregnancy in women in Ngali II (**a**) and Yaoundé (**b**) with known placental malaria status (Ngali II: PM^−^ n = 13, PM^+^ n = 14; Yaoundé: PM^−^ n = 10, PM^+^ n = 15). (a) A significant difference between PM^−^ and PM^+^ women in Ngali II was found throughout pregnancy using multilevel polynomial regression analysis (p = 0.0047). (**b**) PM^−^ women in Yaoundé also had higher mean Ab levels compared to PM^+^ women, although difference was not significant. (**c–d**) Data from PM^−^ and PM^+^ women were subdivided into primigravidae (PG) and multigravidae (MG). All PG in Ngali II and Yaoundé were PM^+^ at delivery, explaining why there are only 3 groups in the figures. Similar Ab levels were present in placental PM^+^ PG and PM^+^ MG. (**e**) The same plasma samples from PM^−^ and PM^+^ women in Ngali II collected at 4 to 7 months were tested for surface binding to CSA-binding IE (7G8 strain). In support of the results in Fig. 2a, higher levels of Ab was detected in women in Ngali II who were PM^−^ compared to those who were PM^+^ (p = 0.05). P values are based on multilevel polynomial regression analysis. Abbreviations: FV2, full-length VAR2CSA; PM^−^, placental malaria-negative; PM^+^, placental malaria-positive; PG, primigravidae; MG, multigravidae.

Data on placental malaria status at delivery were available for 27/39 women in Ngali II and 48/50 in Yaoundé. Characteristics of this subgroup of women did not differ from those in Table 1. Ab levels throughout the course of pregnancy were compared between PM^+^ and PM^−^ women (Fig. 2) using multilevel polynomial regression analysis. In Ngali II, Ab levels increased in a liner fashion (p = 0.0003) in both PM^−^ and PM^+^ women with both groups having similar Ab levels by delivery (Fig. 2a). Notably, PM^−^ women had significantly higher Ab levels throughout the course of pregnancy than PM^+^ women (p = 0.005). Since all PM^−^ women were multigravidae, adjustment could not be made for gravidity in the analysis across groups. However, within the PM^+^ group, there was no significant difference in Ab levels to FV2 between primigravidae and multigravidae (p = 0.54). Therefore, the absence of PM at delivery in Ngali II correlated with Ab levels over time.

In Yaoundé, 23/48 women were PM^−^ and failed to make Ab to FV2 (data not shown). These women had detectable submicroscopic infections or very low parasitemia by microscopy, but they did not produce Ab to FV2. Data from the remaining 25 women who were seropositive for FV2 showed a similar trend to that found in women living in Ngali II. PM^−^ women in Yaoundé had consistently higher levels of Ab compared to PM^+^ women, although the difference in this small group of women did not reach statistical significance (Fig. 2b). Overall, the magnitude of Ab response was lower in women living in Yaoundé.

Placental malaria-negative women at both study sites were exclusively multigravidae (i.e., ≥2 pregnancies). Accordingly, the Ab responses of PM^+^ women were analyzed by gravidity. Longitudinal analysis showed no difference in Ab levels to FV2 between PM^+^ primigravidae (PG) and PM^+^ multigravidae (MG) at both sites (Fig. 2c–d), although PM^+^ MG had slightly higher Ab levels than PM^+^ PG at the earliest time-points in Ngali II. Thus, our data suggest that regardless of transmission intensity, primigravid women are at an increased risk of placental malaria at delivery due to the lack of anti-FV2 Ab prior to 7 months of pregnancy. Even though some primigravidae produced Ab levels comparable to PM^−^ MG by 8 months of pregnancy, the Ab response probably occurred too late to clear parasites from the placenta before delivery. Equally interesting, our data also illustrate that some multigravid women living in either the high or low transmission sites remained susceptible to placental malaria because they did not produce high levels of Ab to FV2 early in pregnancy. Thus, Ab levels late in the 1^st^ through the 2^nd^ trimester appear to be a primary determinant of placental malaria status.

To further dissect when during pregnancy strong Ab responses correlated with absence of placental malaria, mean Ab levels in plasma of women in Ngali II were evaluated at two-month intervals (i.e., 3–4, 5–6, and 7–8 months of pregnancy) with respect to placental malaria status at delivery. Women with intermediate to high anti-FV2 Ab levels (MFI >5,000) at 5–6 months and 7–8 months had 2.3 (95% CI, 1.02–4.94; p = 0.05) and 2.0 times (95% CI, 1.01–3.93; p = 0.10), respectively, decreased risk of placental malaria at delivery compared to women with <5,000 MFI anti-FV2 Ab. In summary, higher Ab levels to FV2 correlated with absence of placental malaria.

### Correlation between Infected Erythrocyte Surface Staining and Absence of Placental Malaria

Ab binding to the surface of placental type IE was evaluated by flow cytometry (Fig. 2e). Antibodies targeting the surface of CSA-adhering IE (7G8 strain) were significantly higher in PM^−^ than PM^+^ women (p = 0.05) (Fig. 2e). The level of surface binding increased linearly from 4 to 7 months in PM^−^ women; whereas, in PM^+^ women levels remained low and only increased during the third trimester. Overall, similar results were obtained in assays measuring Ab to FV2 and binding to the surface of IE.

### Correlation between Percentage of High Avidity Antibodies to Full-length VAR2CSA and Absence of Placental Malaria

**Figure 3:**
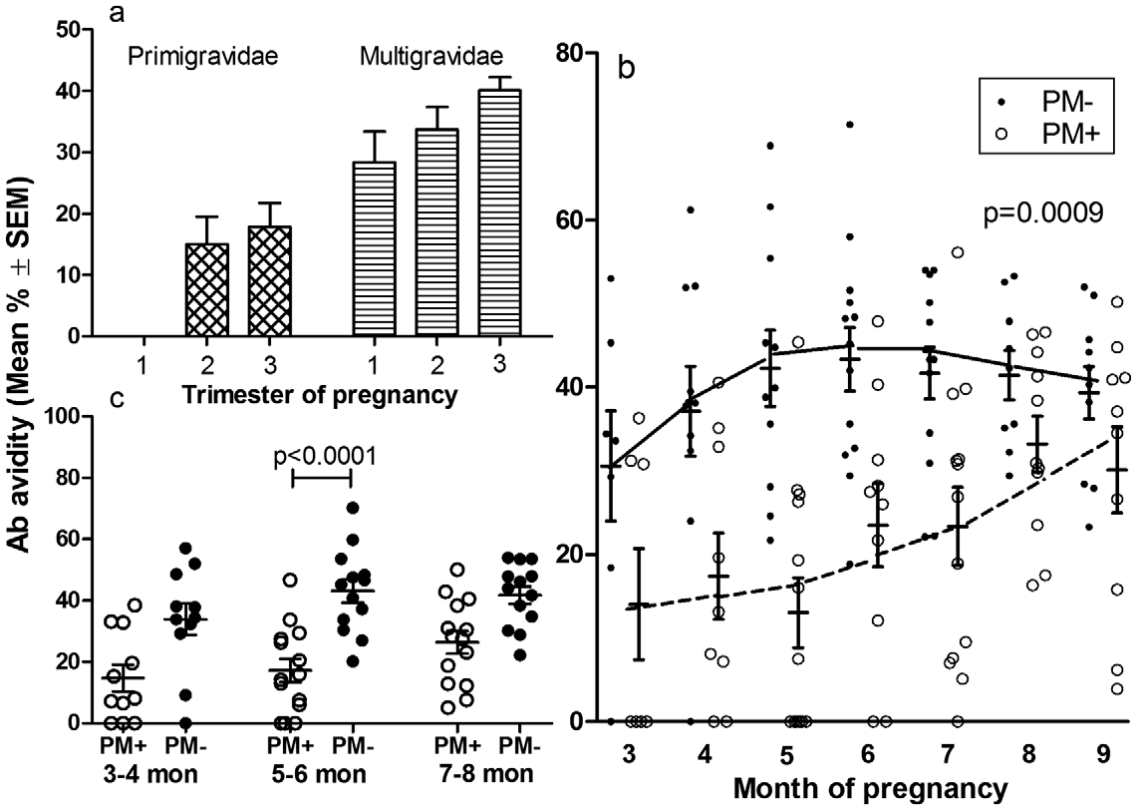
High Avidity Antibodies to VAR2CSA were Associated with Absence of Placental Malaria in Ngali II. (**a**) The proportion (%) of Ab that remains bound to FV2 after incubation with 3M NH_4_SCN (i.e., % high avidity Ab) in the plasma of primigravidae and multigravidae living in Ngali II is shown. Results represent the mean + SEM for 11 to 51 data points per bar. Throughout the course of pregnancy, the proportion of high avidity Ab to FV2 was higher in multigravid than primigravid women (p<0.0001; multilevel polynomial regression analysis). (**b**) The scattergram shows that the proportion of high avidity Ab to FV2 was highly variable. However, the proportion (%) of high avidity Ab in plasma of women who were PM^−^ was significantly higher than in plasma of PM^+^ women during pregnancy (PM^−^, n = 13; PM^+^, n = 14) (p = 0.0009), based on multilevel polynomial regression analysis. (**c**) A significantly higher proportion of high avidity Ab was present in plasma of PM^−^ women than PM^+^ women at 5–6 months (p<0.0001), but not at 3–4 and 7–8 months.

To elucidate if anti-FV2 Ab quality is important in parasite clearance, the proportion of high avidity Ab to FV2 (i.e., Ab remaining bound after treatment with 3M NH_4_SCN) was determined in plasma from women in Ngali II (Fig. 3) and the women in Yaoundé (n = 25) who were seropositive to FV2. This analysis showed that very few women in Yaoundé developed high avidity Ab, preventing further analysis. By comparison, in Ngali II during the first trimester none of the Ab in primigravidae were high avidity. However, by delivery 17.8±3.9% (mean ± SEM) of anti-FV2 Ab were found to be high avidity (Fig. 3a). Furthermore, during the first trimester, 28.4±5.0% of anti-FV2 Ab in multigravidae were high avidity, and this increased to 40.1±2.1% during the third trimester. As expected, the proportion of high avidity Ab was higher throughout pregnancy in multigravidae compared to primigravidae (p<0.0001; multilevel polynomial regression analysis). Thus, the development of high avidity anti-FV2 Ab is gravidity-related. Importantly, the proportion of high avidity Ab was significantly higher in PM^−^ compared to PM^+^ women throughout pregnancy (p = 0.0009) (Fig. 3b). That is, the proportion of high avidity Ab in PM^−^ women rose from 34±12% at 3 months to 42±15% at 5 months, and plateaued at 43% until term. In contrast, in PM^+^ women, only 16±18% of Ab were high avidity at 3 months, 23±16% at 6 months, and 33±12% at the end of pregnancy. Although the proportion of high avidity Ab was generally higher in multigraivd compared to primigravid women, some multigravidae still had placental malaria, suggesting high avidity Ab may be needed early in pregnancy for parasite clearance by delivery. Notably, higher proportion of high avidity anti-FV2 Ab at 5–6 months of pregnancy was significantly associated with absence of placental malaria (p<0.0001). While PM^−^ women still had higher proportion of high avidity Ab at 3–4 and 7–8 months of pregnancy, the differences at these time points were not significant (Fig. 3c). Moreover, women in Ngali II with ≥ 35% high avidity Ab during the 5–6 months of pregnancy had a 7.6 times (95% CI: 1.2–50.0; p = 0.0013) lower risk of placental malaria than women with <35% high avidity Ab. In Yaoundé, the number of women with high avidity Ab was too small for accurate correlation analysis and therefore data are not shown. Thus, at least in the high transmission setting, high avidity Ab to FV2 by the 2^nd^ trimester was found to be important in reducing placental malaria by delivery.

## Discussion

Antibodies that bind to CSA-adhering IE and different DBL domains of VAR2CSA have been associated with improved pregnancy outcomes [8], [10]–[12]. Nonetheless, in previous studies Ab levels at delivery were not found to differ between PM^−^ and PM^+^ women [13], [33]; in fact, higher levels were often associated with active or chronic placental malaria [16], [22]. In the current study, Ab levels to FV2 were significantly higher from early (3–4 months) in pregnancy until term in PM^−^ compared to PM^+^ women (Fig. 2a). Likewise, significantly increased binding of Ab to the surface of IE was observed in PM^−^ women (Fig. 2e). Strong Ab responses to FV2 (i.e., >5,000 MFI) at 5–6 and 7–8 months of pregnancy reduced a woman's risk of having placental malaria 2.3 and 2.0 fold, respectively. Since Ab levels increased during pregnancy (Fig. 1 and Fig. 2), these data help explain the previously reported lack of association between Ab at delivery and absence of placental malaria.

Although the above association was only statistically significant in the high transmission rural village where women were repeatedly infected [34], the same trend was observed in women living in the low transmission urban city. These results agree with our understanding of infection with VAR2CSA-expressing parasites in that Ab responses to VAR2CSA were low or absent early in pregnancy, increased progressively over the course of pregnancy, and were higher following several pregnancies. Overall, these findings suggest that protection from placental malaria correlates with the acquisition of a mature humoral immune response starting during the first half of pregnancy. Previous results showed that the breadth of the Ab response to the 6 DBL domains increases during pregnancy and a broader repertoire was associated with clearance of placental parasites [35]. The current results show that the magnitude and avidity of anti-VAR2CSA Ab continue to evolve over multiple pregnancies and are positively associated with malaria transmission intensity and clearance of placental parasites. Therefore, the overall “quality” of anti-placental malaria antibodies appears to be strongly influenced by malaria transmission intensity.

This study suggests that primigravidae are most likely highly susceptible to placental malaria as they lack high levels of Ab to VAR2CSA during the 1^st^ and 2^nd^ trimesters. Of interest, 66% of primigravid women in Ngali II failed to develop strong Ab responses to FV2 (FCR3 allele), despite being repeatedly infected during pregnancy. In Ngali II, some multigravid women had placental malaria at delivery and they also had lower Ab levels to FV2. Whether this reflects gaps in the antibody repertoire to achieve adequate Ab breadth [35] or additional factors that control immune responsiveness to placental isolates such as parasites having multiple *var2csa* copies [36] should be investigated.

The comparison of women in the city further highlights that malaria transmission intensity has a profound effect on the maturation of the anti-VAR2CSA antibody response. In Yaoundé, only 33% of primigravidae had Ab responses to FV2 at delivery and only 18% of multigravidae developed strong Ab responses to the antigen (Fig. 1). All of the women were either slide- or PCR-positive prior to 6 months of pregnancy, showing that lack of a response was not due to lack of infection (Table 1). It is possible that Ab to other malarial antigens eliminated IE before the parasite expressed the CSA-binding phenotype and sequestered in the placenta, in which case a humoral immune response to VAR2CSA would not have been induced. The combined results from both transmission settings emphasize that development of a vaccine should not only target primigravid women, but women of all gravidities, especially with the changing epidemiological profile of malaria in Africa.

Taken together, our data demonstrate that women who possessed high levels of Ab to FV2, especially those with high avidity, early in pregnancy are likely to be free of placental malaria at delivery. These antibody responses developed better in women from the higher malaria transmission setting, indicating that malaria transmission intensity influences the evolution of protective anti-VAR2CSA antibodies. While we could only use placental malaria status at delivery as a direct outcome measure of Ab to VAR2CSA, it is important to point out that the process of parasite clearance occurred over time during pregnancy. The sooner a woman eliminates malaria parasites from the intervillous space, the better it is for the pregnancy. Thus, vaccines for VAR2CSA should aim to induce high levels of anti-VAR2CSA Ab spanning the entire course of pregnancy. These results are encouraging as they substantiate the important role of anti-VAR2CSA Ab in the clearance of IE from the placenta.

## Materials and Methods

### Study Sites

A prospective cohort study was conducted between 2001 and 2005 in the rural village of Ngali II and in the city of Yaoundé, Cameroon, where malaria transmission is perennial and individuals receive approximately 256 and 13 infectious bites/person/year, respectively [34], [37].

### Study Populations

Women were recruited at government-operated facilities during the first trimester and written informed consent was obtained. At enrollment, malaria histories were recorded and peripheral blood samples collected. Participants were followed monthly, at which time information on their health and anti-malarial drug usage were recorded and peripheral blood samples were collected. Women who became blood-smear positive for *P. falciparum* were prescribed anti-malarial drugs and iron-supplement according to the government policy. At delivery, information on delivery outcome and the neonate was recorded, and samples of maternal peripheral blood, placental blood, and a biopsy of placental tissue were collected for parasitological evaluation. Chloroquine was the first line antimalarial when the study began, but due to increasing resistance, artemisinin in combination with amodiaquine were adopted during the last year of sample collection in the 2004. None of the women were prescribed antimalarial drugs within 5 weeks of delivery. The prevalence of HIV seropositivity was ∼9% at the time of the study. The study was completed before the implementation of intermittent preventive treatment and bednets in Cameroon. All participants gave written informed consent. The study was approved by the National Ethics Committee, Cameroon and the Institutional Review Board, Georgetown University. Use of the coded archived samples and data in the current study was exempted from human subject research by the Committee on Human Studies, University of Hawai'i, Mānoa, USA.

### Diagnosis of Malaria

Blood smears were prepared using maternal blood collected monthly and at delivery. In addition, smears of blood from the intervillous space and impression smears of the placental biopsy were made. Slides were stained with Diff-Quick and read by two microscopists for parasitemia. PCR was used to identify submicroscopic infections [38], [39]. A woman was considered to have placental malaria if IE were detected in placental blood or impression smears.

### Sample Selection

Plasma from all women who met the following criteria were used: information was available on the woman's age, gravidity, and malarial status during pregnancy; confirmed malaria infection detected by microscopy or PCR no later than six months of pregnancy; and ≥4 samples collected throughout pregnancy. In addition, plasma from 20 adult males living at each site was used in the serological assays.

### Expression of Recombinant Proteins

Full-length VAR2CSA was produced in Sf9 insect cells using the baculovirus transfer vector. In brief, the *var2csa* sequence (FCR3 strain, accession number GU249598) was inserted into the Baculovirus transfer vector pAcGP67-A (BD Biosciences) with a histidine tag on the C-terminal end. Linearized Bakpak6 Baculovirus DNA (BD Biosciences) was co-transfected with pAcGP67-A into Sf9 insect cells for generation of recombinant virus particles. Histidine-tagged recombinant protein was purified on Ni^2+^-Sepharose columns from the supernatant of Baculovirus infected High-Five insect cells using an ÄKTA-express purification system (GE-Healthcare). Detailed information on expression and characterization of FV2 protein was previously published [26].

### IgG Antibodies to Full-length Recombinant VAR2CSA

IgG levels to FV2 were measured using the bead-based immunoassay. In brief, saturating amounts (3µg) of recombinant FV2 was covalently coupled to 1 million SeroMAP beads (Luminex Corp.) at pH 7 [40]. The beads were blocked with 1% bovine serum albumin (BSA) in phosphate buffered saline (PBS), pH 7.0 for 24 hr. For Ab measurements, 50µl of plasma diluted to 1∶500 was incubated with 3,000 FV2-coupled beads on a titer plate shaker for 1 hr at room temperature (RT). After washings with 1% BSA-PBS, 100µl of R-phycoerythrin-conjugated affinity purified F(ab')_2_ goat anti-human IgG (Fcγ specific)(Jackson ImmunoResearch) was added and beads were incubated for 1 hr. After washing, median fluorescence intensities (MFI) for ≥100 beads were obtained using a LiquiChip 100 analyzer (Luminex Corp.).

### Percentage of Strong Binding (High Avidity) IgG to Full Length VAR2CSA

We used a modified ammonium thiocyanate (NH_4_SCN) elution protocol [41], [42] and assessed the amount of Ab that remained bound to FV2 after 30 min of incubation with 3M NH_4_SCN (i.e., high avidity Ab). Our approach aimed to determine the proportion of FV2-specific Ab that were of high avidity, rather than the average affinity of the heterogenous Ab pool in each plasma sample. To minimize the effect of Ab concentration on Ab avidity assessment, the proportion of high avidity anti-FV2 Ab was determined using three plasma dilutions spread over the linear range of detection. Then, the mean proportion of high avidity Ab from all three measurements for each sample was determined. In brief, each plasma sample was diluted to 1∶300, 1∶1,000 and 1∶3,000 with 1% BSA-PBS and 50µl of each dilution was incubated with 3,000 FV2-coupled beads as described above. After washing, bead-Ab complexes were incubated for 30 min with 100μl of PBS or 3M NH_4_SCN before R-phycoerythrin-conjugated goat-anti-human IgG (Fcγ specific) was added to beads for 1 hr incubation. Percentage of high avidity Ab was calculated by [MFI beads with NH_4_SCN]/[MFI beads with PBS] x100 and the results from the three dilutions averaged. With this protocol, only a minor variation among the three plasma dilutions was found for the same sample. From assaying over 170 samples, the inter-sample mean standard deviation (SD) is ±4.8% and median SD is ±3.8%.

### Antibody Binding to the Surface of Infected Erythrocytes

The protocol of Avril *et* *al*. was followed [43]. In brief, CSA-binding IE (7G8 strain) were obtained by panning on CSA [44]. Plasma samples were preadsorbed twice with O^+^ erythrocytes. Then, 10 million erythrocytes with 5–8% trophozoites were incubated with a 1∶10 dilution of plasma, followed by FITC-conjugated goat (Fab')_2_ anti-human IgG (1∶100 dilution, PNIM1627, Beckman), evaluated using a LSRII flow cytometer (Becton Dickinson), and data were analyzed using FLOWJO 8.1 software (Tree Star, Inc). Results are expressed as the mean of the adjusted geometric MFI for plasma in duplicate. The adjusted MFI  =  (IEt-UEt) – (IEc–UEc); where IEt  =  MFI of IE incubated with test human plasma; UEt  =  MFI of uninfected erythrocytes incubated with test human plasma; IEc =  MFI of IE incubated with USA control plasma; UEc  =  MFI of uninfected erythrocytes incubated with USA control plasma.

### Statistical Analysis

The Student t-test (unpaired) was used to compare age and length of pregnancy between women in the 2 study sites. Fisher's exact test was used to compare the proportion of women who were slide- and PCR-positive during pregnancy, proportion of Ab responders between sites and the significance of relative risk assessment. FV2 Ab levels and high avidity Ab in PM^−^ and PM^+^ women were analyzed using multilevel polynomial regression analysis. In this analysis, longitudinal FV2 Ab levels and percentage of high avidity Ab were dependent variables, while placental malaria status, month of pregnancy (linear and quadratic), and the interaction between malaria status and time, were independent variables. Results with p values ≤0.05 were considered to be significant.
